# β-Glucan Metabolic and Immunomodulatory Properties and Potential for Clinical Application

**DOI:** 10.3390/jof6040356

**Published:** 2020-12-10

**Authors:** Emma J. Murphy, Emanuele Rezoagli, Ian Major, Neil J. Rowan, John G. Laffey

**Affiliations:** 1 Bioscience Research Institute, Athlone Institute of Technology, N37 HD68 Athlone, Ireland; emurphy@ait.ie (E.J.M.); emanuele.rezoagli@unimib.it (E.R.); nrowan@ait.ie (N.J.R.); 2 Lung Biology Group, Regenerative Medicine Institute at CURAM Centre for Medical Devices, School of Medicine, National University of Ireland Galway, H91 CF50 Galway, Ireland; 3 Anaesthesia and Intensive Care Medicine, University Hospital Galway, H91 YR71 Galway, Ireland; 4 Department of Medicine and Surgery, University of Milano-Bicocca, 20900 Monza, Italy; 5 Materials Research Institute, Athlone Institute of Technology, N37 HD68 Athlone, Ireland; imajor@ait.ie

**Keywords:** β-glucan, clinical trials, biomedicine, immunomodulation, metabolism

## Abstract

β-glucans are complex polysaccharides that are found in several plants and foods, including mushrooms. β-glucans display an array of potentially therapeutic properties. β-glucans have metabolic and gastro-intestinal effects, modulating the gut microbiome, altering lipid and glucose metabolism, reducing cholesterol, leading to their investigation as potential therapies for metabolic syndrome, obesity and diet regulation, gastrointestinal conditions such as irritable bowel, and to reduce cardiovascular and diabetes risk. β-glucans also have immune-modulating effects, leading to their investigation as adjuvant agents for cancers (solid and haematological malignancies), for immune-mediated conditions (e.g., allergic rhinitis, respiratory infections), and to enhance wound healing. The therapeutic potential of β-glucans is evidenced by the fact that two glucan isolates were licensed as drugs in Japan as immune-adjuvant therapy for cancer in 1980. Significant challenges exist to further clinical testing and translation of β-glucans. The diverse range of conditions for which β-glucans are in clinical testing underlines the incomplete understanding of the diverse mechanisms of action of β-glucans, a key knowledge gap. Furthermore, important differences appear to exist in the effects of apparently similar β-glucan preparations, which may be due to differences in sources and extraction procedures, another poorly understood issue. This review will describe the biology, potential mechanisms of action and key therapeutic targets being investigated in clinical trials of β-glucans and identify and discuss the key challenges to successful translation of this intriguing potential therapeutic.

## 1. Introduction

The therapeutic potential of foods containing the polysaccharide beta-glucan (β-glucans) has long been known. Specifically, the medicinal properties of mushrooms—a major source β-glucans—were detailed in manuscripts from India dating back 500 years [1]. A popular mushroom known as *Agaricus blazeii* is native to a small area of the mountains of Brazil near Sao Paulo. More recently, apparent lower incidences of cancers, viral and bacterial-induced illnesses, and increased life spans seen in people living in a small area of the mountains of Brazil near Sao Paulo were attributed by some to the ingestion of this popular local mushroom known as *Agaricus blazeii* [1]. There are at least 700 species of mushrooms like *A. blazeii* that are considered to possess medicinal properties [2,3].

β-glucans are a key active ingredient in mushrooms and are also found in oats, barley, yeast, bacteria and algae. In microbial sources, they are a structural component and in grain sources, they are found in the endospermic and aleuronic walls [4,5,6]. Today, β-glucans are widely marketed as biologically active compounds that have the potential to improve health [7]. Of therapeutic importance, β-glucans have potentially important metabolic and gastro-intestinal effects, modulating the gut microbiome, altering lipid and glucose metabolism, reducing cholesterol, leading to their investigation as potential therapies for metabolic syndrome, obesity and diet regulation, gastrointestinal conditions such as irritable bowel, and to reduce cardiovascular and diabetes risk. β-glucans also appear to have immune-modulating effects, leading to their investigation as adjuvant agents for solid cancers and haematological malignancies, for immune-mediated conditions, such as allergic rhinitis and respiratory tract infections, and to enhance wound healing. Two glucan isolates were licensed as drugs in Japan as an immune-adjuvant therapy for cancer in 1980 [8,9].

Consequently, β-glucans are also being tested for clinical efficacy in clinical trials for a variety of conditions, including inflammatory conditions, cardiometabolic diseases, obesity and cancer. In fact, over 200 clinical trials of β-glucans are completed or in progress, suggesting their diverse biological properties might have the significant therapeutic potential [10]. The US Food and Drug Administration passed a ruling in 1997, stating that oat bran—a major source of β-glucan—was to be registered as the first cholesterol-reducing food, and recommended a dose of 3 g/day of β-glucan, this can be provided by ≤40 g oat bran or ≤60 g oatmeal [11]. However, notwithstanding this ruling, significant challenges exist to the successful clinical testing and translation of β-glucans as a therapy. Our incomplete understanding of the diverse mechanisms of action of β-glucans constitutes a key knowledge gap. A second challenge relates to the important differences that appear to exist in the effects of apparently similar β-glucan preparations, which may be due to differences in sources and extraction procedures, another poorly understood issue. For the most part, β-glucans are marketed as “natural” food (as opposed to medicinal) products, frequently comprising a complex mixture of polysaccharides and even contaminants that can potentially contribute to varied effects and outcomes. [12].

This review will describe the biology, mechanisms of action and key therapeutic targets being investigated in clinical trials of β-glucans. We will also identify and discuss the key challenges to the successful translation of this promising potential therapeutic.

## 2. Discovery and Characterization of β-glucans

In their seminal work on the discovery of Properdin in the complement system, Pillemer and colleagues used a compound called Zymosan [13]. Zymosan is a crude mixture of yeast cell wall materials and was used as an immune stimulator [13]. Zymosan contains polysaccharide proteins, fats, and inorganic elements [14]. Zymosan was also used for a range of applications including the promotion of survival after ionizing radiation, increasing resistance to several biological infections, inhibition of dietary-induced hypercholesterolemia and inhibition of tumour development [14,15,16,17,18].

Zymosan had an abundance of adverse side effects, including pyrexia, anaemia, pulmonary hyperplasia, and microemboli, and so in 1961 Riggi and Diluzio identified the component of Zymosan responsible for reticuloendothelial stimulation (RES) [19]. Their work determined that this stimulatory agent was a polysaccharide from the cell wall of yeast or *Saccharomyces cerevisiae*. This finding was contradictory to previous reports that suggested a lipid mixture in Zymosan was the active ingredient, [15,20]. They concluded that there was a 1,3 β-type linkage that was uniquely characteristic to this polysaccharide or glucan. This linkage is required for the stimulation of cells or RES. This study initiated the era of glucan research [19].

In 1969, Chihara et al. isolated 1,3 1,6 β-glucans form the mushroom *Lentinus edodes.* They demonstrated its ability to inhibit sarcoma in allogenic mice [20]. Since then, β-glucans has been isolated from an abundance of sources, most recently a 1,3 1,6 extract has been isolated from bananas [21]. Two glucan isolates are now licensed drugs in Japan as an immune-adjuvant therapy for cancer since 1980 [8,9]. 

## 3. β-glucan Structure

**Figure 1:**
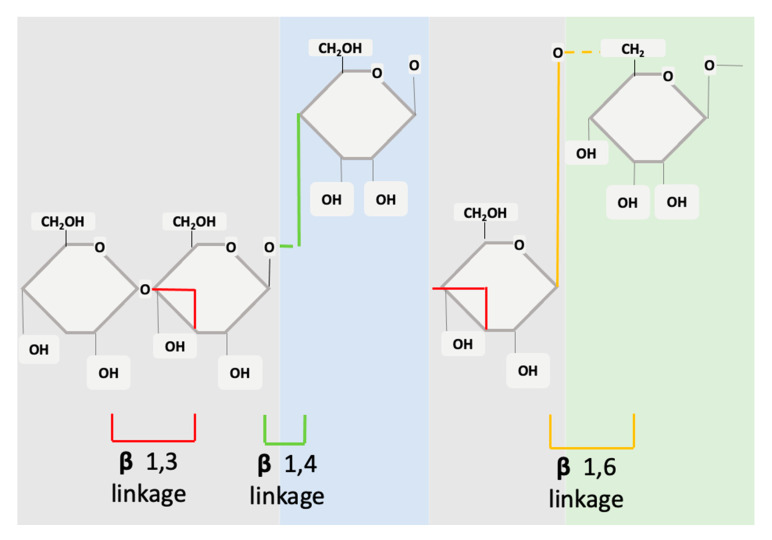
Structure of cereal β-glucans (1,3 1,4) and non-cereal β-glucans (1,3 1,6).

Structurally, β-glucans are comprised of glucose units linked together by several different types of beta-glycosidic linkages (Figure 1). In the basic form, the molecule is a polymer of monosaccharide residues [0]. β-glucans are composed of β-D-glucose monomer units, which are held together by glycosidic linkages at differing positions (1,3), (1,4) or (1,6). This structure can be either branched or unbranched [22]. The β-glucans source will determine if the molecule has branched structures and to what extent. The monosaccharide units interconnect at several points to form a wide variety of different branched and linear structures [23].

The fine structure of β-glucans can vary in meaningful ways that modify its effects and mechanisms of action. A variance will occur between glycosidic linkages, molecular weight, branching, degree of polymerization, and solubility [0,23] β-glucans from different sources will have different effects or functions [24]. Borchani et al. provide a more detailed review of the structural chemistry of β-glucans [25].

When compared to other biopolymers (e.g., proteins), β-glucans have a greater structural variation and therefore have a higher capacity for biological information. This variation and flexibility underlie the potential for β-glucans to influence complicated and diverse cell pathways, activities, and signalling processes [0].

## 4. Sources of β-glucans

**Figure 2:**
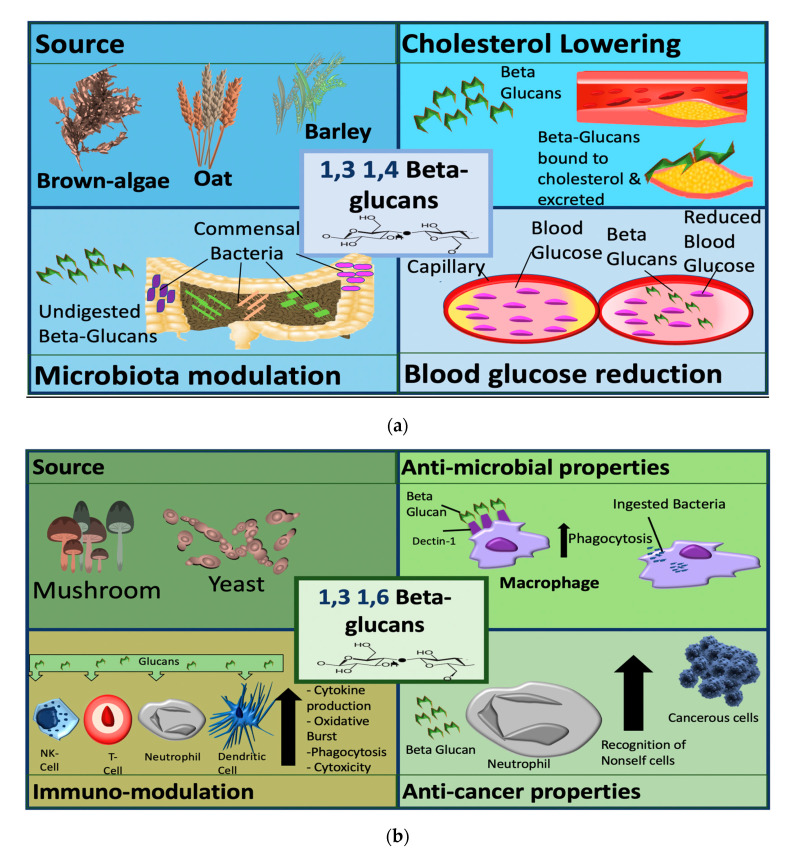
Sources and mechanisms of β-glucans dependent on structure. In the panel (**a**) cereal β-glucans; in the panel (**b**) fungal β-glucans.

β-glucans can be divided into two sub-groups, namely cereal and non-cereal (Figure 2). Cereal or grain derived β-glucans usually have 1,3 1,4 glycosidic linkages without any 1,6 bonds or branching [26,27,28]. They are fibrous structures found in aleurone (proteins stored as granules in the cells of plant seeds), in the sub-aleurone layer and the cell wall of endospores [29]. Cereals include oat, barley, wheat and rice [29].

Regardless of source cereal β-glucans share similar structures, some differences include variation in 1,3 1,4 linkage ratio, molecular size, and some have large cellulose structures [30,31]. β-glucan content also varies among cereal sources—there is higher glucan content in barley then oats, the least is found in rice and wheat [32].

Non-cereal β-glucans are fibrous structures found in yeast, fungi, bacteria, and algae [33]. β-glucans originating from yeast have linear (1,3) backbones with long chains of 1,6 branching [27,34,35]. Unlike grain β-glucans, fungal β-glucans differ between species concerning the degree of branching and distribution [0]. Curdlan, a glucan isolated from *Agrobacterium*, contains no side branching, just a β-D glucan backbone [36]. The solubility of the molecule is reliant on 1,3 linkages. β-glucans are classified as soluble dietary fibre as the beta configuration is not digestible by enzymes in the human gastrointestinal (GI)-tract [37]. They are further classified pharmacologically as biological response modifiers (BRMs) as they influence the immune system counterparts [38]. For cereal-based β-glucan at least, higher molecular weight β-glucans appear to be more effective than lower weight molecules [39]. With this level of variance, it is not surprising that there is a range of diverse applications of β-glucans in clinical trials ranging from the alleviation of respiratory illnesses to improving fatigue and weight loss. 

## 5. Mechanisms of Action

The mechanism of action of β-glucan can be broadly divided into two major areas, namely metabolic/GI effects and immune-modulatory effects. The molecular and structural characteristics will determine functional effects [40,41,42]. β-glucans display diverse mechanisms of action and have a defined structure-activity relationship [43].

### 5.1. Role of β-glucans Structure

A distinguishing characteristic of all β-glucans that is necessary for biological activity is its 1,3 backbone [44,45]. The degree and the specific profile of biological activity appear to be related to specific β-glucans structural characteristics. First, side-chain frequency and length are important, with a higher degree of branching associated with greater biological activity [46]. Structures with a branching frequency of 0.20 and 0.33 appear to be the most biologically active [45,47]. While it remains unclear precisely how differences in structure modulate the activity of β-glucans [48], sidechains length and frequency play a crucial role in the immunomodulatory activity [48,49]. The overall size of the β-glucans molecule is also important, with higher molecular weight fractions having a greater effect [48,50].

### 5.2. Role of β-glucan Source

β-glucans are classified by their source, into cereal and non-cereal β-glucans (Figure 2). Cereal β-glucans, which are 1,3 and 1,4 linked, mainly display metabolic activities, such as the ability to lower cholesterol and blood glucose and have been explored in clinical studies to target metabolic conditions. These 1,3 and 1,4 linked glucans appear to be recognized as dietary fibres after ingestion and elicit their metabolic effects via this mechanism. Non-cereal (predominantly fungal and yeast) β-glucans have more pronounced immunomodulatory functions and are the focus of immunomodulation and anti-cancer studies. Fungal and yeast β-glucans have a 1,3 and 1,6 linkage structure and are recognized by some receptors including dectin 1, complement receptor 3 (CR3) and toll-like receptors (TLRs) [51,52,53,54].

The pathways activated by β-glucans are not fully understood, but β-glucans appear to be recognized as pathogen-associated molecular patterns (PAMPs) and they modulate immune cell function via this mechanism. The different mechanisms of β-glucans dependent on the structure are categorized by source in Figure 2.

β-glucans from the same source can also differ in activity profile. In work carried out by the authors, we demonstrated that two β-glucan extracts from the same mushroom could have different effect profiles in an in-vitro lung injury model [55].

### 5.3. Cholesterol-Lowering Effects

Coronary vascular disease is directly correlated with metabolic syndrome [56]. Metabolic syndrome is characterized by a cluster of metabolic dysfunctions—including abdominal obesity, atherogenic dyslipidaemia, small LDL particles and low HDL cholesterol levels, elevated blood pressure, insulin resistance and glucose intolerance [57] (Table 1). Cholesterol plays a key role in the pathogenesis of plaque formation. While cholesterol at normal levels is beneficial to the body, higher levels leads to the accumulation of cholesterol deposits in the blood vessel walls—termed atherosclerotic plaques—which lead to blockage of arteries supplying key organs including the heart [58]. The enzyme 3-hydroxy-3-methyl glutaryl-co-enzyme A (HMG-CoA) reductase acts as a catalyst in the production of cholesterol.

The synthesis of bile acids occurs in the liver, in a series of reactions that convert hydrophobic cholesterol into a water-soluble compound, thus representing the primary pathway for cholesterol catabolism [59,60]. The formation of primary bile acids is primarily catalysed by the cytochrome P450 enzyme 7-α hydroxylase (CYP7A1) [61]. CYP7A1 regulates cholesterol synthesis as well as its excretion from the liver [62,63,64]. Bile acids are directly involved in the regulation of cholesterol [65]. Barley beta-glucan has been shown to regulate CYP7A1 and HMG-CoA and ultimately regulate cholesterol synthesis and its decomposition into bile acids. By regulating enzyme activity in the liver, cholesterol is reduced in the blood vessels [64].

β-glucans may also elicit some of their effects on cholesterol through modulation of the gut microbiota, which is a key regulator of bile acid metabolism [66]. As β-glucans are resistant to the digestion of gastric and pancreatic enzymes they are fermented in the colon by host microbiota and elicit their effects in this way. There have been reports of the correlation of changes to microbiota and reduction of total cholesterol after β-glucan administration [67]. Oat and barley β-glucans are fermented by intestinal microflora resulting in SCFA as the end products [68]. When the SCFAs are absorbed they inhibit cholesterol synthesis by limiting the activity of HMG-CoA or through the catabolism of LDL-cholesterol [64]. Other research groups have suggested that foods containing β-glucans may influence the gut microbiota with ultimate effects on bile acid signalling and SCFA signalling which regulate cholesterol metabolism [66].

Cereal β-glucans also increase faecal bile acid excretion [69]. A preclinical study in pigs demonstrated that glucans in the diet increased bile acid and cholesterol metabolism, this study also showed the potential prebiotic effect on gut microbiota [70] (please see the section below on “Effects on Gut and microbiota”).

The cholesterol-lowering effects of cereal β-glucans appear to be correlated to their viscosity. [71,72]. Highly viscous fibres such as β-glucans have demonstrated viscosity-dependent health benefits including cholesterol-lowering and improved glycaemic control [73]. A clinical trial that examined the physicochemical properties of β-glucans found that cholesterol-lowering ability was correlated to viscosity, the high-viscosity preparations displayed the strongest effect. [74].Viscous β-glucans also appear to modulate host bile acid metabolism [73]. β-glucan can bind to bile which is produced in the liver, and increase faecal excretion ultimately decreasing cholesterol build-up [75]. More specifically, the β-glucans bind the whole micelles that contain bile acids in the intestine. This prevents interaction with luminal membrane transporters that are located on the intestinal epithelium, preventing the absorption of fats and cholesterol [76,77]. This ultimately lowers systemic LDL cholesterol as a result of the reduction of de novo synthesis of bile acids from cholesterol [73].

In a clinical trial in 268 men and women with high cholesterol, oat β-glucans reduced cholesterol and triglyceride, lowering the risk of cardiovascular diseases [78]. Other studies have shown that oat β-glucans reduce systolic and diastolic blood pressure and thus improve patients’ blood pressure when administered daily. Oat β-glucans have also reduced the risk of cardiovascular disease in hypertensive patients. [77,79,80,81].

### 5.4. Enhancement of Glycaemic Control

Cereal derived β-glucans decrease insulin resistance [82] and reduce postprandial blood glucose concentrations [72,83,84,85,86,87,88]. Products containing oat β-glucans have been shown to decrease postprandial glucose in healthy individuals [80,89,90] and type II diabetic patients [91,92]. A recent CT has found significant correlations between oat β-glucans viscosity, glucose and insulin levels and gastric emptying responses. The study showed that oat β-glucans favourably slowed down gastric emptying and reduced glycaemic and insulinemic responses in healthy individuals [93]. This study also demonstrated that viscosity would determine the effect of the β-glucans. It is hypothesized that by forming viscous solutions and retaining water in the digestive tract stool volume is increased and homeostasis of blood glucose levels are improved [37,94].

A study by Miyamoto et al. 2018 investigated the metabolic effects of Barley flour containing varying amounts of β-glucan, in a preclinical mouse model of high-fat diet-induced obesity. Barley glucan caused appetite suppression and improved insulin sensitivity via short-chain fatty acid (SCFA) -induced production of gut hormones. High levels of β-glucan decreased fat gain which improved insulin sensitivity [95]. In pigs, 6% of oat b-glucan significantly decreased glucose concentrations and increased insulin levels. It is hypothesized that these changes were associated with gastric emptying peptide and GLP-1 [96].

In terms of mechanism of action, viscous β-glucans can form a gelatinous layer in the gut, which acts as a barrier and hinders absorption of glucose and lipids [97,98,99]. The layer acts as a filter and slows digestion and absorption, larger molecules are not filtered through and pass directly through to the intestine [100]. Other hypotheses are that the layer delays starch interaction and thus reduction in the absorption of carbohydrates and ultimate reduction of glycaemia [101].

There are some apparent controversies and conflicts in the literature regarding the glycaemic effects of β-glucans. These relate in part to the extrapolation of findings from one specific glucan preparation to other preparations from the same source, despite the fact that cultivation methods and extraction procedures will all influence the activity profile. In addition, β-glucans can establish secondary structures which can also affect activity [48]. For example, barley β-glucan has shown positive effects on glucose control levels in rats when fed diets containing barley β-glucan over twelve weeks and in another study where rats were fed diets containing barley β-glucan over six weeks [102,103]. In contrast, when barley was administered to obese rats for two weeks, there was no effect on postprandial glycaemic response. In this study, both a high concentration and a low concentration barley β-glucan was administered. The authors state that their study did not influence glucose levels. It must be highlighted that the authors used two novel barley varieties [104]. As the structure is unknown, this extract cannot be compared to other barley extracts or can conclusions be made about treatment durations as it is likely that each experiment used a different extract.

### 5.5. Effects on Gut and Microbiota

Oat β-glucan also acts as a prebiotic improving gastrointestinal function indirectly by enhancing the intestinal microbiota given that is a non-digestible by humans [105]. It stimulates the growth and activity of commensal bacteria in the colon [106]. The growth of normal intestinal microbiota (*Lactobacilli* and *Bifidobacteria* species) are supported by β-glucans in-vivo and in-vitro models [68]. When rats were administered oat and barley glucan, they demonstrated higher *Lactobacilli* and *Bifidobacteria* availability. These experiments showed that higher doses of glucan had a better effect and oat glucan was more effective over barley [107,108]. Other species of intestinal microbiota enhanced by β-glucans include *Lactobacillus acidophilus*, *Lactobacillus casei* and *Bifobacterium* spp. [109].

The regulation of various pathways and digestion is improved when the human microbiome is enriched [110], such as by prebiotic like β-glucans. The microbiota also helps to breakdown non-digestible β-glucans into SCFAs which can display biological activity [111], such as influencing hormone secretion. Specifically, the improved insulin sensitivity may result from the promotion of gut hormone secretion from enteroendocrine cells by SCFAs [95]. SCFA stimulated gut hormone secretion from enteroendocrine cells has also been shown in human studies [112].

### 5.6. Key Knowledge Deficits

Unfortunately, our understanding of β-glucan structure-activity and source-activity relationships remains limited. The effect of one β-glucan preparation is frequently extrapolated to all β-glucans [48], which is an over-simplification as multiple factors affect the structure and activity of each β-glucans preparation. Insufficient attention is being given in research publications to the importance of glucan source or of variations in glucan structures to their activity profile, with less than 20% of 10,000 publications relating to immunological activities of β-glucans, including structure in the title or abstract in one analysis [48]. An array of effects is being attributed to β-glucans that in reality are likely to vary substantially depending on the originating glucan source and preparation method, which both have significant effects on glucan structure and hence on activity profile.

Additional research is therefore required to fully understand and characterize the key mechanisms of bioactive and metabolic activity of β-glucans. It is critically important that research carried out in this field describes clearly the source, molecular weight and molecular structure of the specific β-glucan molecule being investigated. Methods that can be potentially employed for this purpose include structural analysis; nuclear magnetic resonance (NMR), chemolytic methods (methylation analysis), vibrational spectroscopic methods (Fourier transformed infrared spectroscopy FTIR, Raman) and Gel permeation chromatography methods (GPC) for molecular weight determination [113,114]. It is only when this is addressed will key source-structure-function relationships of β-glucans be properly established.

## 6. Fungal β-glucans (1,3 and 1,6 Linked)

Fungal β-glucans, such as those isolated from fungi (e.g., mushrooms) and yeast appear to have a more immune-modulating effect profile, leading to their investigation as priming or activation adjuvant agents for infectious diseases or cancers (Table 2). β-glucans from yeast have been shown to activate the immune response and initiate the inflammation process as well as improving resistance to infections and inhibiting cancer development [115]. Of importance, β-glucans do not have a direct cytotoxic effect on cancerous cells or tumours, but instead, elicit an indirect effect through the activation of immune cells [8].

Mushroom derived β-glucans are the most potent anti-tumour and immune-modulating of the β-glucans [0,45]. Mushroom β-glucans have demonstrated positive therapeutic effects in respiratory conditions, preventing recurrent respiratory tract infections in children [116], prevention of symptoms of allergic rhinitis, and upper respiratory tract infections [117,118].

β-glucans appear to alleviate allergic problems to allergens e.g., pollen via mechanisms related to the decreasing pro-inflammatory cytokines IL-6, TNF-α and increased formation of anti-oxidants [119,120]. Immune cells recognize β-glucans as PAMPs. Thus, glucan effects are elicited through pattern recognition receptors (PRRs). These include Dectin-1, Complement receptor 3 (CR3), Toll-like receptors (TLRs), Lactosylceramides, scavenger receptors [121,122,123]. Dectin-1 is the critical receptor for β-glucans [124]. TLR 2,4 and 6 co-bind to dectin-1 after glucan recognition [125]. This recognition and binding of TLR and dectin-1 modulate the release of pro-and anti-inflammatory cytokines to control the immune response [126].

The exact mechanism by which β-glucans suppress inflammatory cytokines and induce anti-inflammatory cytokines are complex and incompletely understood. In fact, non-cereal β-glucans inhibit lipopolysaccharide-induced nitric oxide and TNF-α release in-vitro. [127], and reduce the secretion of TNF-α, IL-6 in lipopolysaccharide challenged mice [128]. Stimulated monocytes isolated from glucan treated mice release less TNF-α and IL-6 after toxic stimulation [129]. Mushroom derived β-glucans reduced pro-inflammatory cytokine levels in healthy female volunteers [130].

When β-glucan binds to dectin-1, TLRs are also required in this recognition for the release of inflammatory cytokines [131,132,133]. Perhaps during injury one of these receptors is blocked, or during injury, the β-glucans bind to a separate receptor [134]. β-glucans from yeast have been shown to induce a strong immune-modulatory cytokine interleukin 1-receptor antagonist (IL-1Ra) expression. This expression is independent of the common β-glucan receptors (Dectin-1 and CR3). Smeekens et al. suggest that an unknown β-glucan receptor exists that specifically induces an Akt/P13K-dependant anti-inflammatory response [134]. Our previous work demonstrated that β-glucans from Shiitake mushroom, induced NFKβ in-vitro in the absence of injury. In the presence of LPS, the same extract significantly reduced injury [135].

Other research areas of interest about the use of β-glucans for clinical purposes were summarized in Table 3.

## 7. Insights into β-Glucan Effects from Pre-Clinical Models

### 7.1. Bacterial Sepsis

β-glucans improved the immune response and survival of mice from influenza infection [136]. In a model of *E. coli* injury (intraperitoneal injection), bacterial counts in peripheral blood reached zero in mice administered β-glucans, while mortality in control animals was 100% at 24 h [137]. PGG is a commercial source of purified yeast β-glucans that has been shown to enhance bacterial clearance from blood and reduce mortality in rat intra-abdominal sepsis models [138,139,140]. PGG enhanced clearance of antibiotic-resistant *S. aureus* in a rat intra-abdominal infection model, potentially mediated by increasing circulating monocytes and neutrophils and increasing neutrophil oxidative microbicidal activity without generating harmful inflammatory responses. PGG treatment also had a synergistic activity with antibiotic administration to further enhance bacterial clearance and reduce infection-related mortality [141].

In a rat model of caecal ligation puncture (CLP) induced polymicrobial sepsis, β-glucan treatment attenuated pro-inflammatory cytokines TNF-α and IL-6 elevations, while increasing anti-inflammatory cytokine IL-10 concentrations and accession of cellular antioxidants ultimately protecting the cells from oxidative stress [142].

### 7.2. Lung Injury

β-glucans decreased lung injury severity following abdominal aortic ischemia-reperfusion in rats, reducing oxidative stress, decreasing lung permeability (reduced alveolar protein concentration and wet/dry lung ratio), reduced the systemic inflammatory response, decreased lung leukocyte infiltration, and decreased histologic evidence of lung injury [143]. β-glucans decreased lung injury severity following CLP induced sepsis [144]. β-glucan treatment decreased circulating monocytes and lymphocytes in bronchoalveolar lavage and reduced secondary lung injury as a result of CLP induced sepsis. β-glucans decreased inflammatory cytokines TNF-α, IL-1β and IL-6, and reduced the lung injury score, in a lung injury model induced by CLP [145].

### 7.3. Cancer Therapy

One of the most interesting applications of β-glucans is for cancer treatment, specifically as an adjuvant to enhance “conventional” cancer chemotherapeutics. β-glucans regulate complement-dependent cytotoxicity (CDC). β-glucans are recognized as PAMPs, triggering the response of immune effector cells. This will then elicit an anticancer immune response through the formation of a complex. When they enter the bloodstream, they are bound by endogenous plasma anti-β glucan antibodies (ABA). This binding activates complement and complement protein iC3b binds to the ABA, resulting in β-glucans—ABA—iC3b complex [146,147]. This complex binds to immune effector cells and activates specific aspects of innate immune function including CR3 phagocytosis. The activation and formation of this complex facilitate the direct killing of antibody-targeted tumour cells [146,147].

This mechanism was observed in both a pre-clinical model [148] and demonstrated with whole blood from healthy volunteers. The authors found that anti-cancer properties are dependent on the formation of the complex with naturally occurring ABAs [146]. In the preclinical model, mice were administered anti-tumour monoclonal antibodies (mABs) in combination with β-glucans. Results showed that the dual treatment produced significantly greater tumour regression in both mammary and hepatic tumours. The combinational treatment had a greater effect on each treatment individually. Interestingly, mice that were deficient in CR3 or serum CR3 or granulocytes did respond to treatment [148].

## 8. Clinical Trials of β-glucans

This review focused on the current clinical trials (CTs) registered on clinicaltrials.gov. The CTs were identified by an electronic search using the keyword “glucan”. The search was last performed on 23rd November 2020. CTs that used β-glucans for diagnostic purposes for fungal infections were excluded as were the trials using glucose polymers not specifying glucans as treatment. We identified over 200 registered clinical trials of β-glucans on clinicaltrials.gov. Trials that included measuring elevated blood β-glucan levels as a diagnostic test for fungal infections, or that used glucose polymers instead of β-glucans, are excluded from this analysis. In CTs, β-glucans have been administered orally in capsules, in food and as part of vaccines as adjuvants. Studies have shown that oral administration is as active as the injected dose [149]. The majority of studies for intervention purposes were aimed at oral administration of (cereal-derived) β-glucan for metabolic diseases (Table 1).

**Figure 3:**
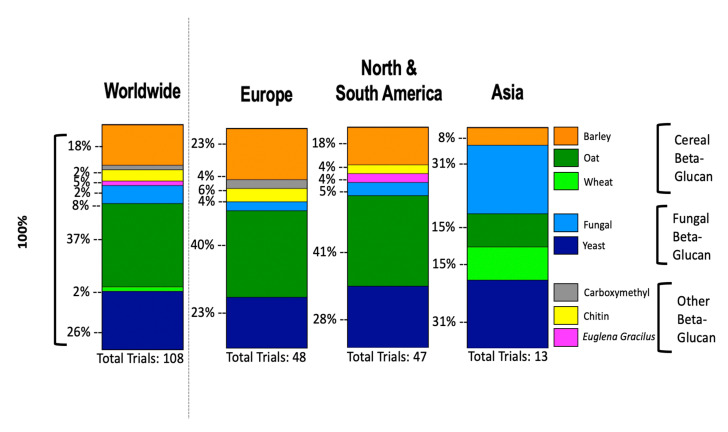
Bar graphs displaying the percentage of beta-glucan source used worldwide (**left panel**), further stratified for the three continents of Europe, America and Asia (**right panel**). Data obtained from clinicaltrials.gov.

**Figure 4:**
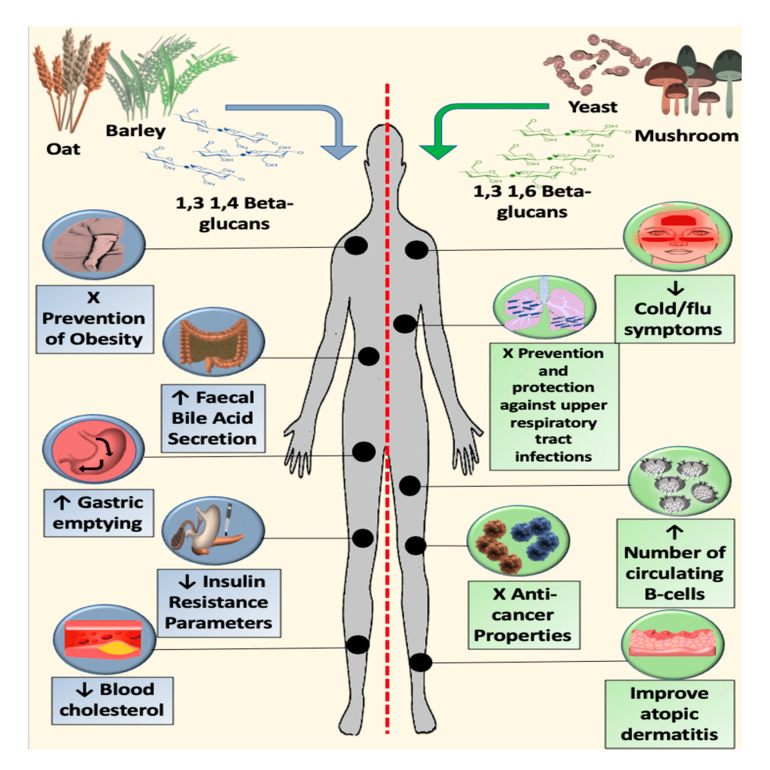
Graphical representation of the effects of two different structures of beta-glucan in clinical trials.

We reported the use of β-glucan by source worldwide and stratified by 3 main continents (Figure 3) and we represented an image that summarizes updated applications of β-glucan in clinical trials (Figure 4).

## 9. Metabolic Effects of Cereal β-glucans

When barley containing β-glucan ranging from 3 g to 5 g was administered to 30 mildly hypercholesterolemic patients in a controlled, four-phase, crossover trial copositive effects included an increase of faecal bile acid excretion and SCFAs [150], reduction of blood cholesterol levels [151], prevention and treatment of obesity [152], reduction in visceral fat obesity [153], the influence of gastric emptying and glycaemic response [154].

In clinical studies, reported benefits of oat β-glucan include beneficial modulation of postprandial glycaemia [155] and satiety [156], improved appetite control [157], reduction of serum LDL cholesterol [74,158,159] reduction in total cholesterol [158], reduction of inflammation and oxidation hypercholesterolemic patients [160], and reduction in insulin-resistant parameters [161].

A clinical trial evaluated the safety of pre-treatment of 1,3 1,6 β-glucan in patients undergoing coronary artery bypass grafting (CABG) found the treatment was well tolerated. The measured parameters also showed that the isoenzyme creatine kinase was significantly reduced. Release of this enzyme is associated with muscle damage [162]. A study carried out in 1991 investigated the cholesterol-lowering potential of β-glucan in the diet. At six weeks, significant differences were observed for both total cholesterol and LDL in the β-glucan treatment group [163].

β-glucans were shown to reduce a 50% bile acid secretion from the small bowel in patients who were administered oat bran. This effect was not observed when glucanase enzymes were used to hydrolyse the β-glucan [164].

Patients with mild to moderate hyperlipidaemia were administered a low dosage diet of 3 g/day of β-glucans. This dose did not significantly reduce total cholesterol or LDL cholesterol [165]. Patients with elevated cholesterol given 6 g of concentrated oat glucan per day, demonstrated a significant reduction in total cholesterol or LDL cholesterol [159].

The study by Velikonja et al. 2019 (NCT02041104) aimed to determine if the consumption of 6 g/day of barley-derived β-glucans (given in bread) could modify gut microbiota composition, production of short-chain fatty acids, and improve metabolic status in patients with metabolic syndrome. The β-glucans were concentrated from barley with dry milling, sieving, and air classification technology. The barley β-glucan significantly lowered cholesterol levels. β-glucans decreased triglyceride levels and reduced subject weight. The treatment surprisingly decreased gastrointestinal bacterial diversity and richness. Test group participants also noticed an increase in stool frequency and flatulence [166].

Wang et al. investigated the physiochemical relationship between β-glucans and their ability to lower cholesterol (NCT01408719). Their objective was to determine if both the molecular weight and/or the daily dose of β-glucan affected cholesterol. Secondly, they wished to determine if genetic variations in genes (CYP7A1 and APOE) associated with cholesterol metabolism influenced the responsiveness of serum cholesterol biomarkers to β-glucan. β-glucans were obtained from barley using food processing protocols, micronizing, boiling, or toasting. The doses administered were 3 g/day or a higher dose of 5 g/day. Results demonstrated that the physicochemical properties of the β-glucan molecule affect activity. The higher molecular weight β-glucans lowered total cholesterol, while the lower molecular weight β-glucans did not. Interestingly, they showed that individuals carrying the G allele of the CYP7A1 gene were more responsive to the higher molecular weight β-glucans’ ability to lower cholesterol in comparison to the participants homozygous for the T allele [167].

Other clinical trials have investigated the effects of β-glucans indirectly. An example of this is the trial registered NCT00069524. This study investigated the anti-hyperlipidaemic effects of oyster mushrooms, which are rich in β-glucans. The trial assessed the safety and efficacy of oyster mushrooms in patients with HIV and antiretroviral treatment-induced hyperlipidaemia. Participants were administered 15 g/day of freeze-dried mushroom. However, the mushroom did not lower non-HDL cholesterol in the participants [168].

## 10. Immunomodulatory Effects of Fungal β-glucans

β-glucans originating from yeast or fungal sources are most widely recognized for their immunomodulatory effects, although other β-glucans may possess similar if less potent effects. Jensenak et al. 2013 investigated the potential immunomodulatory and preventative clinical effect of mushroom derived β-glucans in combination with vitamin C in children with recurrent respiratory tract infections. Although the treatment resulted in a significant reduction in respiratory morbidity as well as a reduction in the number of flu-like symptoms and infections, the β-glucan alone cannot be attributed to these effects as it was administered in combination with Vitamin C [116]. Another similar CT enrolled children with acute rhinopharyngitis and recurrent respiratory infections. Participants were administered resveratrol plus carboxymethyl-β-glucan or saline isotonic solution. The treatment significantly reduced nasal obstruction, rhinorrhoea, sneezing, cough, fever and use of medications [169]. However, the β-glucan therapy was also administered in combination therefore the positive effects observed cannot solely be attributed to β-glucan administration. In contrast, β-glucans from Shiitake mushroom did not affect immune parameters in healthy subjects, except for an increase in the number of circulating B-cells. The treatment was safe and well-tolerated [170].

Trauma patients exhibit increased susceptibility to infection, in a randomized double-blind placebo-controlled trial 38 patients trauma patients undergoing surgery were administered β-glucan of the unknown source [171]. β-glucan (50 mg/m^2^) was administered intravenously daily for seven days. Results showed that morbidity from sepsis was significantly greater in the placebo group (49%) compared to β-glucan group (9.5%). There was a positive correlation between β-glucan administration, immune function measured using IL-1β and decreased septicaemia.

A randomized double-blind controlled CT treated patient with severe multiple trauma with β-glucan (312–685 mg/patient) intravenously to prevent nosocomial pneumonia and sepsis. Interestingly, pneumonia occurred in 11 out of 20-patients in the control group and only two patients in the β-glucan treatment group. The mortality rate related to infection in the control group was found to be 30% and 4.8% in the treatment group. The overall mortality rate that included cerebral deaths was 42% in the control group and 23% in the treatment group [172].

A series of CTs carried out by Babineau et al., commenced with a randomized Phase I/II double-blind placebo-controlled study administering PGG β-glucan to high-risk patients undergoing major abdominal or thoracic surgery. Patients were administered 0.5 mg/kg of PGG 12 to 24 h preoperatively. There were no adverse drug reactions observed. Results demonstrated that treated patients had significantly fewer infectious complications, decreased intravenous antibiotic requirement and shorter ICU days. Molecular parameters and cytokine were not measured therefore the mechanism cannot be determined. However, measurement of leukocyte function of treated patients showed increased killing towards *S. aureus* and *Candida albicans* in vitro. These results were not statistically significant. Limitations of this study included a small study size of 30 patients [173]. The second CT carried out by Babineau and colleagues, was an interventional, multicentre, double-blind, randomized, placebo-controlled study. High-risk patients undergoing major thoracic or abdominal surgery were administered saline or PGG at increasing doses (0.1 mg/kg, 0.5 mg/kg and 1 mg/kg or 2 mg/kg). One dose was administered preoperatively, and a further three doses were administered postoperatively. Results showed that there were reductions in infection incidences among patients who received 0.5 mg/kg of PGG in comparison to the placebo group and the group administered the lowest concentration of PGG 0.1 mg/kg. Only one patient who received 0.5 mg/kg developed a severe infection. Diabetic patients who received the higher doses (0.5 mg/kg, and 1mg/kg or 2 mg/kg) had significantly lower incidences of infection in comparison to patients who received the lowest dose or placebo groups. Patients who were administered 0.5 mg/kg had fewer hospitalization days [174].

The final CT in this series enrolled 1249 patients in a multicentre, prospective, randomized double-blind placebo-controlled trial. Patients enrolled were scheduled for gastrointestinal procedures with two or more defined risk factors. Patients in treatment groups were administered PGG glucan at a dose of 0.5 mg/kg or 1.0 mg/kg preoperatively and three times postoperatively. Results showed that there was no difference in severe infections and mortality between treatment and placebo groups. In malnourished patients undergoing noncolorectal procedures, PGG reduced postoperative infection and death. Unfortunately, the study was ultimately terminated due to patients experiencing more frequent adverse reactions in the treatment group compared to control [175]. Leentjens et al. (NCT01727895) investigated the effects of orally administered β-glucan on innate immune responses in humans. Test groups received 1000 mg/day of a water-insoluble commercial β-glucan from yeast (glucan #300). For analysis, peripheral blood mononuclear cells (PBMCs) were collected and cultured. Cells were treated with various stimuli, and ELISAs were performed. A microbicidal activity assay and β-glucan detection assay were also performed. Results showed that β-glucan was not detectable in serum, and the immune response was not modulated or enhanced [176], suggesting that perhaps the dose used was sub-therapeutic.

Hala Helmi Hazzaa and colleagues in their clinical trial (NCT02402296) investigated the potential immune activation of β-glucans in dentistry. The focus of this trial was to identify a potential substance that would stimulate protective immune responses and influence mount pathways that would contribute to resolving chronic lesions observed in periodontal disease. Results showed that test groups had a higher mean of probing pocket-depth reduction, and a reduction in gingival inflammation compared to the control group. Protective healing patterns were also enhanced. The β-glucan administered was called Imurrill commercial capsules, the source unknown. Publication of this data in a peer-reviewed journal is awaited.

## 11. CTs Cancer Therapy

β-glucans are widely studied as a potential cancer treatment both alone and in combination with other chemotherapeutics and mAb vaccinations. Lentinan a purified polysaccharide from the mushroom Shiitake was administered to patients with advanced or recurrent stomach, colorectal and breast cancers. The extract was administered intravenously at 1 mg/day or 2 mg/day. The extract was also administered with two chemotherapeutic compounds (5-FU and Tegafur). Life span prolongation was measured and a significant effect was observed on host immune response, although this cannot be attributed to Lentinan as it was administered in combination with two other drugs [177].

In patients with myelodysplastic syndromes, Maitake mushroom extract increased neutrophil and monocyte function. Monocyte response to *E. coli* was also reduced. The treatment was well tolerated [178]. Maitake extract was used, thus β-glucans cannot exclusively be responsible for these effects. Another CT administered β-glucans to patients with advanced malignancies receiving chemotherapy. The patients were monitored for tolerability and to determine any effects on haematopoiesis. The treatment was well tolerated with some amelioration of the blood counts but a larger trial is required for confirmation [179].

In a Phase I CT which enrolled 20 patients with chronic lymphocytic leukaemia, monoclonal antibodies in combination with PGG glucan was administered as a treatment. In this study, the combination treatment was well tolerated and therefore administered as a treatment in the follow-on Phase II study. Monotherapy with rituximab and alemtuzumab specifically rarely achieved a complete response or a sustained response. The concept of this study was that the β-glucans would increase the cytotoxic capacity of the innate immune system when administered with the mAb. The authors stated that the study was too small to provide preliminary data, but there was some promise with this application as the addition of β-glucans did improve the duration of response of mAb [180]. In support of this concept is another CT where the same PGG glucan was administered in another mAb trial for the treatment of small-cell lung cancer (NSCLC) [181]. The combination improved efficacy improved antitumor antibody therapy and improved objective response rate (ORR) in enrolled patients. In another study, the same patient group (NSCLC) were enrolled, this time administered mAb, β-glucans and chemotherapeutics demonstrating promising results [182]. At present β-glucans formulations in these trials are being developed for the treatment of cancer in conjunction with tumour targeted antibodies. The Phase I/II trial NCT03003468 is currently underway due to be completed in September 2021. This trial will investigate PGG, the mAb pembrolizumab in patients with NSCLC. The use of β-glucans as a potential cancer treatment is promising as it seems to be well tolerated in patients. However, as the majority of these studies administer β-glucans in combination with anticancer drugs or as part of monoclonal antibody treatment, attributing any effect specifically to β-glucans will be difficult.

## 12. Translational Challenges and Opportunities

The wide range of variety within the β-glucan class of macromolecules may explain their lack of translation to clinical use despite their promising mechanistic effects. The diversity between β-glucan samples occurs physiochemically in regard to conformation (inter-molecular and intra-molecular forces), degree of branching, monosaccharide composition, linkage ratio and linkage type. Also to consider are co-extracted chemicals such as mannose which can dilute or even contaminate β-glucan therapeutics [183]. It is established that molecular weights have a direct effect on activity. There is variance in molecular weights between sources with extraction procedure also having influence [184,185,186,187]. β-glucans originating from the same species can also have a range of molecular weights. For example, β-glucans can have a range of molecular weight values ranging from 65–3100 × 10^3^, 31–2700 × 10^3^, 21–1100 × 10^3^, and 209–487 × 10^3^ for oat, barley, rye and wheat [185]. Ultimately, this diversity amongst samples produces inconsistent results as essentially every research group is using a different product.

A potential solution to this is to isolate β-glucan using standardized (openly reported) methodologies, from the best-understood sources (barley, oats, mushrooms) characterize the molecular effects of a carefully selected range of these β-glucan samples (low and high degree of branching, low and high molecular weight; one plant and one fungal source) and then focus on the translation of the most promising of these β-glucan variants. In this way, research groups can compare results more precisely. Another solution to variance amongst biological samples is a chemical or enzymatic modification of the samples. However, this approach may have disadvantages such as lowered activity and potential toxicity [187].

The translational approach to β-glucan is characterized by heterogeneity in application also. Specifically, routes of administration, dose, time points, populations and length of treatment all vary in preclinical and clinical applications. This approach has led to apparently conflicting findings [188].

In another study, lower doses of β-glucan increased body weight and elevated plasma glucose and triglyceride levels. In this study, the higher doses presented more beneficial effects [95]. Furthermore, β-glucans display limited weak solubility under neutral conditions and unsuitable hydrophobic/hydrophilic balance [187]. Molecular modifications of polysaccharides can increase water solubility but this, in turn, may affect biological activity, the introduction of potentially harmful chemicals. This instability will ultimately influence the route of administration.

There is no standardized method for β-glucan extraction which leads to huge variances in preparations. Most importantly, very few articles state the amount of β-glucan in each product or the method by which they determined this. Structural variability, low purity levels and unknown receptor pathways all contribute to the current limitations of β-glucan research. The physiochemical differences between preparations which include molecular weight, degree of branching, solubility, denaturing affect receptor binding. This leads to the activation of multiple pathways with no key pathways being defined.

Specific targeting of cereal-derived β-glucan (with their 1,3 1,4 branching pattern) to metabolic disorders and microbial/fungal derived β-glucan (with their 1,3 1,6 branching pattern) to immune-modulatory indications might best harness the different effect profiles of both glucan sources. Similar approaches could be taken to differing molecular weights of glucans, to best define their optimal indications within these general areas. This raises the possibility that cereal glucans may eventually be better classified as a dietary supplement while non-cereal β-glucan might find therapeutic immune-modulatory uses as an active pharmaceutical ingredient.

Finally, disease targets studies to date remain very broad and the target mechanism of action is generally not clearly defined. Focusing on elucidating the most therapeutically relevant mechanisms of action, and on developing better characterized b-glucan preparations, may lead to the successful translation of b-glucans for a number of focused metabolic and immune-modulatory indications for which the most promising evidence exists.

## 13. Conclusions

β-glucans are natural molecules that have significant therapeutic promise, particularly as metabolic and immune-modulatory agents [189]. Enthusiasm for their therapeutic potential is reflected in the high number of clinical trials of β-glucans that have been completed or are in progress. However, concerns around deficits in the understanding of the complex relationship between β-glucans structure and their effect profile, together with heterogeneity in the approach to clinical translation, and variations in the approaches to extracting and purifying these agents, has hampered the search for clinical indications for β-glucans. A more rigorous approach, carefully defining optimal isolation and purification procedures from key sources, then characterizing the relationship between specific variations in a β-glucans branching structure, molecular weight, and cereal/microbial source and their effect profile may be the best approach to ultimately realize the therapeutic promise of these intriguing compounds. Thus, facilitating a greater understanding of the mechanisms of action of β-glucans. This could lead to targeting a more precise clinical objective as the mechanism of action would be established.

## Figures and Tables

**Table 1:** Cereal glucans in metabolic/GI disorders.

Areas of Research	Study Title	NCT	Design	β-glucan Type	Dose	Population
**Microbiota and gastrointestinal health**	Characterization of the Gut Microbiota Composition and Activity After Three Weeks of Chitin-glucan Supplementation	NCT03505177	n/a	Chitin-glucan	4.5 g/day	Healthy
	Characterization of Chitin-glucan Fibre Fermentation in Human After a Single Administration	NCT03494491	n/a	Chitin-glucan	4.5 g/day	Healthy
	Effect of 6 Weeks Daily Consumption of a Cereal-based Juice Beverage on Gastrointestinal Health (NEWDRINK)	NCT03046667	n/a	Barley	Drink = 1 dose per day—dose not stated	Irritable Bowel
	Characterization of Gut Microbiota Composition and Activity After a Daily Supplementation of 4.5 g/Day of Chitin Glucan Fibre during 3 Weeks in At-cardiometabolic Risk Volunteers (FITACHITIN)	NCT03773900	n/a	Chitin	1.5 g/3 times daily	Cardiometabolic Risk Abdominal Obesity
	β-glucan on Faecal Microflora in Polypectomised Patients	NCT00893659	n/a	Unknown	3 g/day	Polypectomised Patients
	Synbiotics and Gastrointestinal Function Related Quality of Life After Colectomy for Cancer	NCT01479907	n/a	Unknown	2.5 g/sachet	Colorectal Neoplasms
	The Effect of Oats Containing 1.4g β Glucan on Faecal Bacterial Population(s) and Plasma Cholesterol in Healthy Adults with Elevated Cholesterol Levels	NCT03450395	n/a	Oats	40 g of crude oats/day	Microbiome Plasma Cholesterol Prebiotic
	Beta-glucan Effects on Lipid Profile, Glycemia and intestinal Health (BELT) (BELT)	NCT03313713	n/a	Unknown	3 g/day	Atherosclerosis
	Healthy Effects of an Innovative Probiotic Pasta (SFLABPASTA)	NCT02236533	n/a	Barley	Pasta once a day	Obesity, Inflammation, Dyslipidaemia, Constipation
	The Effectiveness of Pleuran in Treatment of Acute Gastroenteritis in Children (EPTAGE)	NCT03988257	Phase 2	Mushroom	10 mg Pleuran	Diarrhoea; Acute
	Impact of Consumption of Beta-glucans on the Intestinal Microbiota and Glucose and Lipid Metabolism	NCT02041104	n/a	Barley	6 g/day	Metabolic Syndrome, Dyslipidaemia, Obesity, Abdominal, Hyperglycaemia, Hypertension
	Combined Nutritional Therapies for the Treatment of Ulcerative Colitis	NCT03444311	n/a	Oat	Unknown	Colitis, Ulcerative
	Prebiotic Supplementation and Intestinal Barrier Function in Elderly: an RCT	NCT03336385	n/a	Oat	Daily—dose not known	Prebiotics, Aged
	Chronic Cardiovascular and Gut-bacteria Effects of Phenolic Rich Oats in Adults with Above Average Blood Pressure	NCT02847312	n/a	Oat	60 g of Oat cake	Healthy
	The Effect of Hot Cereal on Digestive Health in Children	NCT02868515	n/a	Oat	3 g/day	Subjective Measures of Digestive, Health Post Consumption
	Dietary Fibres Effect on the Gut Microbiota Composition	NCT04114513	n/a	Unknown Tate & Lyle powder	2 increasing to 8 g per day	Microbiome, Metabolic Syndrome, Cardiovascular Risk Factor, Inflammation, Dyslipidaemias
	β -1,3/1,6-D-Glucan Ganoderma Lucidum on Ulcerative Colitis	NCT04029649	Phase 2, Phase 3	Fungal	Capsule containing 180 mg/three times daily	Ulcerative Colitis
**Glycemic control; Diabetes**	β glucan and Acetate Production	NCT03714646	n/a	Unknown	12 g once	Pre-Diabetes, Obesity
	Inulin and Acetate Production and Human Substrate Metabolism	NCT03711383	n/a	Unknown	Unknown	Obesity, Pre-Diabetes
	Efficacy and Safety Study of Soluble Beta-1,3/1,6-glucan (SBG) Versus Placebo in Chronic Diabetic Foot Ulcers	NCT00804414	Phase 3	SBG—Yeast	Topical Application	Diabetes, Diabetic Ulcer
	The Glycaemic Response Elicited by β-glucans of Different Physical Properties and Form	NCT01610518	n/a	Oat	4 g	Type 2 Diabetes
	Effect of Serving Size and Addition of Sugar on the Glycaemic Response Elicited by Oatmeal (Panther)	NCT02506972	n/a	Oat	30 g of oats	Diabetes Mellitus
	Effect of Viscous Soluble Fibres on Body Weight	NCT03257449	n/a	Oat, Barley	Unknown	Overweight and Obesity, T2DM (Type 2 Diabetes Mellitus), General Population
	Impact of DHA/Oat on Metabolic Health in Gestational Diabetes Mellitus	NCT03569501	n/a	Oat	4.05 mg/day	Gestational Diabetes Mellitus in Pregnancy
	Efficacy of Soluble β-1,3/1,6-Glucan Compared to Placebo on Chronic Leg Ulcers in Diabetes Patients	NCT00288392	Phase 2	SBG—Yeast	Unknown	Foot Ulcer
	Food Modification to Alter Glycaemia and Insulinaemia	NCT03706378	n/a	Yellow Noodle—wheat	50 g of β-glucan in 230.4 g of yellow noodle per day	Diabetes
	The Glycaemic Response of Local Foods Using the Continuous Glucose Monitoring System	NCT03703544	n/a	Yellow Noodle—wheat	Unknown	Diabetes
	Oat β-glucan as a Supplement in Chilean Type 2 Diabetics	NCT04299763	Phase 2	Oat	5 g daily with breakfast	Type2 Diabetes
	The Effect of Insoluble Yeast β-glucan Intake on Pre-diabetic Patients	NCT03495362	n/a	Yeast	500 mg capsule twice a day	Pre-diabetic
	Effects of (1,3), (1,6)- β-D-glucan on Insulin Sensitivity and Inflammatory Markers of the Metabolic Syndrome	NCT00403689	n/a	Yeast	1.5 g/daily	Overweight
	Intake of Beta-glucan and Postprandial Regulation of Blood Glucose Metabolism in Healthy Subjects	NCT03293693	n/a	unknown	0.5 g–8 g	Post Prandial Blood Glucose, Gut Microbiota, Satiety
	The Effect of Content of Barley Beta-glucans in Bread on Postprandial Blood Sugar (ARRS-bGL-01)	NCT03878576	n/a	Barley	25 g	Glycaemic Index
	Evaluation of Woulgan in Diabetic Foot Ulcer	NCT02631512	Phase 4	Woulgan-contains SBG—yeast	Gel Application	Diabetic Foot Ulcers
	A Study of the Effect of Oats on Post Prandial Glucose Response	NCT02651597	n/a	Oat	Unknown	Normoglycemic, Normal Body Weight
	Effects of Barley on Glucose Control	NCT02367989	n/a	Barley	2–6 g/day	Healthy
	Barley and Rice Mixture Effects on Blood Glucose	NCT03387345	n/a	Barley	Unknown	Blood Glucose, Dietary Fibre, Hunger
**Lipid Regulation**	Effect of the Molecular Weight of Oat β-glucan on Its Ability to Lower Serum Cholesterol (Bluebird)	NCT00981981	Phase 2	Oat	3–4 g/day	Hypercholesterolemia
	Effect of Beta-glucan on Cholesterol Lowering	NCT01408719	n/a	Barley	3–5 g β glucan	Hypercholesterolemia
	The Effect of Viscous Dietary Fibres on LDL-cholesterol	NCT04133805	n/a	Barley, Oat	Unknown	Cardiovascular Risk Factor, Hypercholesterolemia
	Oat and Cholesterol	NCT03911427	n/a	Oat	Powdered sachets three times daily	Elevated LDL Cholesterol
	Impact of Consumption of Oats in Lipid Profile of Children and Adolescents with Dyslipidaemia	NCT01581697	Phase 1, Phase 2	Oat bran	3 g with 3 meals a day	Atherosclerosis, Hypercholesterolemia
	Nutritional Counselling Associated with the Ingestion of Oat Bran in Hypercholesterolemic Subjects	NCT02189200	n/a	Oat	40 g oat bran per day	Dietary Modification
	Effects of Lentinula Edodes Bars on Dyslipidaemia and Oxidative Stress in Cholesterol Individuals: Randomized Study	NCT04186780	n/a	Fungal	2 cereal bars of Shiitake per day	Dyslipidaemias
	Effects of Chitin-glucan on Oxidized Low-Density Lipoprotein (LDL)	NCT01232309	n/a	Chitin	1.5 g–4.5 g of glucan	Cardiovascular
**Obesity and diet regulation**	Effects of Oligofructose and Barley on Satiety and Energy Intake	NCT00776256	n/a	Oats, Barley	1 g/serving	Appetite, Regulation
	β-glucan and Insulin Sensitivity in Obese Humans	NCT01393210	n/a	Unknown	Unknown	Obesity
	Diet for the Maintenance of Weight Loss and Metabolic Health in Obese Postmenopausal Women (WELCOME)	NCT04136093	n/a	Oat, Barley	50 g of oatmeal and barley groats	Metabolic Syndrome, Diet Modification, Postmenopause
	Efficacy of Hydroxycinnamates and Beta-glucans as a Dietary Tool Against Obesity Pilot Study (OBHEALTH_PS) (OBHEALTH_PS)	NCT04321590	n/a	Oat	3 g or 5 g/day	Overweight, Obesity
	Dietary Fibres and Satiety in Bariatric Patients (FIBAR)	NCT03573258	Early phase 1	Oat	6 g	Bariatric Surgery Candidate
	SATIN: Satiety Innovation. Study 2-University of Aberdeen (SATIN)	NCT02604316	n/a	Viscofibre, Oat and Barley	6 g for 10 days	Overweight, Obesity
	Effects of β-glucan on Energy Intake and Satiety	NCT02637388	n/a	Oatwell, Powder—Oats	4 g as part of breakfast	Obesity
	A Trial Comparing a Diet Including Products Aimed at Targeting Satiety (SATIN)	NCT02485743	n/a	Unknown	Unknown	Weight, Appetite
	The Effect of a Breakfast Meal Containing Oat β-glucan on Food Intake at a Subsequent Meal in Normal-weight and Overweight Subjects	NCT03490851	n/a	Oat	2–4 g	Satiety
	Efficacy and Safety of Fermented Barley on Decrement of Body Fat in Obese Subjects	NCT01402128	Phase 2, Phase 3	Barley	3 g/day	Overweight; Hyperlipidaemia
	The Effect of Dietary Fibre on Food Liking	NCT03241238	n/a	Unknown	Unknown	Different Fermentable Fibre, Satiation
**Metabolic Syndrome**	β-glucans and the Metabolic Syndrome—a Human Intervention Study Under BEST	NCT01317264	n/a	Oat, Barley, Mutant Barley	5 g/day	healthy
	Effect of Dietary Fibre and Whole Grain on the Metabolic Syndrome	NCT01316354	n/a	Unconfirmed	Bread with 50 g available carbohydrate	Metabolic Syndrome
	Pivotal Assessment of the Effects of Bioactive on Health and Wellbeing. From Human Genome to Food Industry (PATHWAY-27)	NCT02702713	n/a	Oat (N.C), Pathway-27 website http://www.pathway27.eu/	3 g beta-glucan—not stated frequency	Metabolic Syndrome
	Metabolic Effect of New Foods Through Gut-brain Axis (CHECKMATE)	NCT01851304	n/a	Barley	3 g/100 g bread portion	Obesity, Overweight
	Effects of β-glucan From Barley and Oats on Glucose and Lipid Metabolism, and satiety (glucan)	NCT03648112	n/a	Barley, Oats	80 g crude flakes/day (oat or barley)	Lipid Metabolism, Glucose Metabolism, Satiety
	Effects of Dried Bilberry, Liquid Oats, or Their Combination After AMI (BIOAMI)	NCT03620266	n/a	Glucanova^®—^Oat	Shakes containing oats 3 times daily,	Myocardial Infarction
	Influence of Dietary Fibre-rich Meals on Gene Expression and Postprandial Glucose and Lipid Response	NCT01005342	n/a	Oat	62–82 g—single intake	Hypoglycaemia, Hyperglycaemia
	The Effects of β-glucan Enriched Oatcake Consumption on Metabolic Disease Risk Factors	NCT02615444	n/a	Oat	4 g/day	Metabolic Syndrome X, Cardiovascular Diseases
	Canola Oil, Fibre and DHA Enhanced Clinical Trial	NCT02091583	n/a	Barley	3 g/day	Metabolic Syndrome
	Glycaemic Impact of Oatmeal Plus OatWellXF28	NCT02818452	n/a	Oat	27 g–32.72 g of Oatmeal containing β-glucan	Glycaemic Responses
	Four-hour Glycaemic Kinetic Response Following 13C-enriched Oatmeal Breakfast Compared to Hot Corn Grits	NCT03165773	n/a	Oat	87 g oatmeal	Glycaemic and Insulinemic Response
	Portfolio 5—Multicentre Dietary Advice on Serum Lipids in Hyperlipidaemia	NCT00438425	n/a	Oat, Barley	9.8 g/1000 kcal	Hyperlipidaemia, Cardiovascular Disease
	Magnetic Resonance Imaging-Portfolio Diet Study #7 (MRIPD#7)	NCT02078635	n/a	Oat, Barley	9.8 g/1000 kcal	Cardiovascular Diseases, Hypercholesterolemia, Diabetes, Metabolic Syndrome, Obesity
	Effects of Chitin-glucan on Oxidized Low-Density Lipoprotein (LDL)	NCT01232309	n/a	Chitin	1.5 g–4.5 g daily	Cardiovascular
	Compare the Efficacy and Safety of β-glucan as Add-On to Statin in Subjects with Hyperlipidaemia. (BetAvena)	NCT03857256	Phase 2	CP105F, Oat β-glucan	1.5 g, 3 g or 6 g daily, Administered 3 times daily	Hyperlipidaemias
	The Effect of Oral β-glucan Supplement on Appetite and Insulin Resistance in non-Alcoholic Fatty Liver Disease	NCT02178839	n/a	Oat	3 g daily	Non-Alcoholic Fatty Liver Disease
	ProAliFun_6.5_Health Effects of a Functional Pasta Enriched with Barley Beta-glucans on Healthy Subjects (ProAliFun65)	NCT02710513	n/a	Barley	100 g of β-glucan pasta/day	Healthy
	Clinical Trial to Evaluate the Addition to an Antiretroviral Treatment of a Probiotic (RECOVER)	NCT03542786	n/a	Oat	As part of pro-biotic once a day for 6 months	HIV, Premature Aging

**Table 2:** Fungal β-glucans for immunomodulatory indications.

Areas of Research	Study Title:	NCT	Design	β-glucan Type	Dose	Population
**Solid cancer and Haematological malignancy**	Efficacy and Safety Study of SBG vs Placebo in Head and Neck Cancer Patients Undergoing Radiation Therapy	NCT00790322	Phase III	Soluble- β-glucan SBG, Yeast derived	Not-stated	Head and Neck Cancer
	The Protective Effect of Soluble Beta-1,3/1,6-glucan Compared to Placebo in Oral Mucositis in Head and Neck Cancer Patients	NCT00289003	Phase II	SBG—Yeast	Unknown	Oral Mucositis
	Safety of Soluble β-glucan (SBG) in Treatment of Patients with Non-Hodgkin’s Lymphoma	NCT00533728	Phase 1	SBG, Yeast	Unknown	Non-Hodgkin’s Lymphoma
	Effect of SBG in Patients with Breast Cancer	NCT00533364	Phase 1, Phase 2	SBG, Yeast	Unknown	Breast Cancer
	The Effect of β-glucan in Non-Small Cell Lung Cancer	NCT00682032	n/a	Imucell WGP-Yeast	1 (one) 250 mg β-glucan capsule 3 times a day for 14 days	Non-Small Cell Lung Cancer
	Bivalent Vaccine with Escalating Doses of the Immunological Adjuvant OPT-821, in Combination with Oral β-glucan for High-Risk Neuroblastoma	NCT00911560	Phase 1, Phase 2	Yeast	Oral β-glucan (40 mg/kg/day) in conjunction with vaccine	Neuroblastoma
	β-glucan and Monoclonal Antibody 3F8 in Treating Patients with Metastatic Neuroblastoma	NCT00492167	Phase 1	Yeast	In conjunction with monoclonal antibody—dose-escalation study of β-glucan	Neuroblastoma
	Lung Cancer Vaccine Plus Oral Dietary Supplement	NCT01829373	Phase 1	Vaccine plus oral beta-glucan-Yeast	Unknown	Lung Cancer
	β-glucan and Rituximab in Treating Young Patients with Relapsed or Progressive Lymphoma or Leukaemia, or Lymphoproliferative Disorder Related to Donor Stem Cell Transplantation	NCT00087009	Phase 1	Unknown	Oral β-glucan in conjunction with IV Rituximab	Leukaemia, Lymphoma, Lymphoproliferative Disorder
	Rituximab Plus β-glucan in Chronic Lymphocytic Leukaemia (CLL)/Small Lymphocytic Lymphoma (SLL)	NCT00290407	Phase 2	Imucell WGP, Yeast	250 mg, orally (tablet), three times a day for 9 weeks	Leukaemia, Lymphocytic, Chronic, Lymphoma, Small Lymphocytic
	β-glucan and Monoclonal Antibody in Treating Patients with Metastatic Neuroblastoma	NCT00037011	Phase 1	Unknown	Oral β-glucan in conjunction with IV antibody	Neuroblastoma
	β-glucan in Treating Patients with Locally Advanced or Metastatic non-Small Cell Lung Cancer	NCT00857025	Phase 1	glucan MM-10-001, Fungal	Oral β-glucan once daily	Lung Cancer
	Imprime PGG, Alemtuzumab, and Rituximab in Treating Patients with High-Risk Chronic Lymphocytic Leukaemia	NCT01269385	Phase 1, Phase 2	PGG β-glucan, Imprime PGG, Yeast	Dose escalation study IV administration	B-cell Chronic Lymphocytic Leukaemia, Refractory Chronic Lymphocytic Leukaemia, Stage 0 Chronic Lymphocytic Leukaemia, Stage I Chronic Lymphocytic Leukaemia, Stage II Chronic Lymphocytic Leukaemia
	(PM-01) IMPRIME PGG^®^ With BTH1704 and Gemcitabine for Advanced Pancreatic Cancer (PM-01)	NCT02132403	Phase 1	IMPRIME PGG, Yeast	Assigned doses	Pancreatic Cancer
	A Phase 2 Clinical Trial of Rituxan and B-glucan PGG in Relapsed Indolent Non-Hodgkin Lymphoma	NCT02086175	Phase 2	IMPRIME PGG, Yeast	I.V 4 mg/kg weekly for 4 weeks.	Relapsed/Refractory Indolent B Cell Non-Hodgkin Lymphoma
	Biological Therapy in Treating Patients with Neuroblastoma That Has Not Responded to Previous Treatment	NCT00089258	Phase 2	Unknown	Unknown	Neuroblastoma
	MucoLox Formulation to Mitigate Mucositis Symptoms in Head/Neck Cancer	NCT03461354	Phase 2	Unknown	Unknown	Mucositis Oral, Head and Neck Cancer
	Phase 2 Study of Imprime PGG & Pembrolizumab in Subjects with Adv SCCHN Who Failed Pembro Monotherapy or Experiencing SD	NCT03246685	Phase 2	Imprime PGG, Yeast	4 mg/kg IV over a 2-h infusion time on Days 1, 8 and 15 of each 3-week treatment cycle.	Squamous Cell Carcinoma of the Head and Neck
	Study of Imprime PGG and Pembrolizumab in Advanced Melanoma and Triple-Negative Breast Cancer	NCT02981303	Phase 2	Imprime, PGG, Yeast	4 mg/kg IV over a 2-h infusion time	Advanced Melanoma, Triple-Negative Breast Cancer
	Phase I, Dose-Escalation Study of Soluble Beta-glucan (SBG) in Patients with Advanced Solid Tumours	NCT01910597	Phase 1	SBG -yeast	Unknown	Advanced Solid Tumours
**Immunomodulation**	Efficacy and Safety of Resveratrol and Carbossimetyl Beta-glucan in Treatment of Upper Airways Disease in Infancy (VIRNEO)	NCT03683108	Phase 3, Phase 3	Carbossimetyl β glucan, Carbossimetyl β glucan	3 drops, 4 times a day for 1 week.	Common Cold
	Nebulized Resveratrol Plus Carboxymethyl-β-glucan for Reducing IL-5 in Children with Allergic Rhinitis (RENIM)	NCT03349619	Phase 4	Carboxymethyl-β-glucan	Two sprays (100 uL/spray) three times a day for 4 weeks	Allergic Rhinitis
	Effects of Orally Administered Beta-glucan on Leukocyte Function in Humans (BG)	NCT01727895	n/a	Glucan, #300, Yeast	500 mg/day	Immunologic Deficiency Syndromes
	Safety and Efficacy Study of Oral XIGO Tablets to Treat Common Cold	NCT01092039	n/a	Unknown	Unknown	Common Cold
	Efficacy and Safety of Imuneks 10 mg Capsules in the Prophylaxis of Cold	NCT02807220	Phase 4	Micro ionized β-glucan -source unknown	10 mg—2 capsules every morning	Cold Symptoms
	Alleviation of Cedar Pollen Induced Allergic Symptoms by Orally Taken Superfine β-1,3-glucan	NCT00276445	Phase 4	Fungal	Unknown	Allergic Conjunctivitis

**Table 3:** Other effects of β-glucans.

Areas of Research	Study Title	NCT	Design	β-glucan Type	Dose	Population
**Wound Healing**	Efficacy and Safety Study of Soluble β-1,3/1,6-glucan in Thermal Burns	NCT00283426	Phase 1	SBG, Yeast	Unknown	Burns
	A Randomized Comparison Study of Aquacel Ag and Glucan II as Donor Site Dressings	NCT00581217	n/a	Glucan II, Oat	unknown	Burns
	Multi-Centre, Prospective, Randomized, Comparison of AWBAT™-D vs. Xeroform™ or Glucan II™ for Treatment of Donor Sites in Burn Surgery (AWBAT-D)	NCT00964470	n/a	Glucan II, Oat	Unknown	Treatment of Donor Site Burns
	Efficacy of TR 987, β-1,3-1,6-D-glucan, in the Treatment of Chronic Venous Insufficiency Ulcers	NCT03154619	Phase 2	Glucoprime, Yeast	Gel application twice weekly	Venous Leg Ulcer
	Preadmission Skin Wipe Use for Surgical Site Infection Prophylaxis in Adult Orthopaedic Surgery Patients	NCT03401749	Phase 4	Unknown	Ingredient in wipes before surgery	Surgical Site Infection
	To Study the Effect of β-glucans on Wound Healing	NCT02078128	n/a	Unknown	30 mg/kg daily	Burns
	Beta-1,3/1,6-D-glucan Ganoderma Lucidum on Non-infectious and Idiopathic Uveitis	NCT04162314	Phase 2, Phase 3	Fungal	Capsule containing 180 mg/three times daily	Uveitis
	Soluble β-glucan (SBG) as Treatment for Diabetic Foot Ulcers	NCT00632008	Phase 3	SBG—Yeast	Topical Application twice a week	Chronic Diabetic Foot Ulcers
	Clinical Trial to Evaluate Papilocare^®^ Gel Efficacy into Repairment of Cervical Lesions Caused by HPV (PAPILOCAN)	NCT04210336	Phase 3	Unknown	Topical application	HPV Infection, Lesion Cervix
	Treatment of Chronic Anal Fissure (TOCA)	NCT02158013	n/a	Yeast	Gel application/ twice daily two weeks	Chronic anal fissure
	Irritation and Anal Bleeding in Patients Affected by Haemorrhoids.	NCT03569930	Phase 4	Unknown	Anal application Frequency, Unknown	Haemorrhoids
**Cognitive Performance**	A Follow-up Trial of Proglucamune^®^ in the Treatment of Protective Qi Insufficiency, a TCM Condition	NCT03782974	n/a	Yeast	2 tablets of proglucamune/day—200 mg of β-glucan for 8 weeks	Protective Qi Insufficiency (a Condition Term From TCM)
	An Evaluation of Proglucamune in the Treatment of Protective Qi Insufficiency	NCT03829228	n/a	Yeast, Fungal, Proglucamune tablet	2–100 mg tablets per day	Protective Qi Insufficiency
	Investigation of How Morning Nutrition Influences Cognitive Performance	NCT03169283	n/a	Oat (NC)	Cereal β-glucan in morning	Cognitive Performance
**Safety Studies**	Dose Escalation Safety Study of MM-10-001 in Healthy Subjects	NCT00677027	Phase 1	Lentinan—Fungal	Unknown	Healthy
